# Volcano‐like Activity Trends in Au@Pd Catalysts: The Role of Pd Loading and Nanoparticle Size

**DOI:** 10.1002/cphc.202500164

**Published:** 2025-07-03

**Authors:** Adriano H. Braga, Jhonatan L. Fiorio, Ofelia Yang, Karla L. C. Silva, Tiago A. Silva, Adam S. Hoffman, Simon R. Bare, Jefferson Bettini, Naga Vishnu V. Mogili, Liane M. Rossi

**Affiliations:** ^1^ Departamento de Química Fundamental Instituto de Química Universidade de São Paulo Cidade Universitária Av. Prof. Lineu Prestes 748 São Paulo São Paulo 05508‐000 Brazil; ^2^ Stanford Synchrotron Radiation Lightsource SLAC National Accelerator Laboratory Menlo Park California 94025 USA; ^3^ Brazilian Nanotechnology National Laboratory (LNNano) Brazilian Center for Research in Energy and Materials (CNPEM) Campinas São Paulo 13083‐100 Brazil; ^4^ School of Technology State University of Campinas (UNICAMP) Limeira São Paulo 13484‐332 Brazil

**Keywords:** alloys, gold, nanoparticles, oxidation, X‐ray absorption spectroscopy

## Abstract

The addition of palladium (Pd) to preformed gold nanoparticles (Au NPs) enables the formation of core‐shell structures with enhanced catalytic performance in oxidation reactions. However, predicting the precise palladium content required to achieve maximum catalytic activity remains difficult based on current understanding. Herein, Pd was systematically introduced onto titania‐supported Au NPs (2, 6, and 10 nm) to evaluate their performance in benzyl alcohol oxidation. A volcano‐like trend in catalytic activity was observed, where activity increased with Pd addition, peaked, and then declined. The Pd loading required for maximum activity depended on Au NP size: ≈40 at% Pd/Au for 2.6 nm, ≈20 at% Pd/Au for 6.4 nm, and ≈12.5 at% Pd/Au for 10.6 nm. For Au NPs > 6 nm, peak activity aligned with monolayer Pd coverage, while for smaller NPs (2–3 nm), optimal Pd content was below monolayer predictions. X‐ray absorption spectroscopy revealed a core‐shell structure at low Pd content, but higher Pd loadings led to Pd diffusion into the Au core. This structural transformation likely caused activity decline, indicating that AuPd alloying negatively impacts catalysis. These results highlight that core‐shell Au@Pd catalysts outperform AuPd alloys and provide crucial insights for designing highly active bimetallic catalysts.

## Graphical abstract

The catalytic activity of gold in benzyl alcohol oxidation is enhanced by adding palladium, as long as it remains on the surface in a core‐shell configuration. Palladium migration to the gold core (alloy formation) decreases activity, as shown by the volcano plot. Each gold core size requires a specific Pd amount for optimal activity.

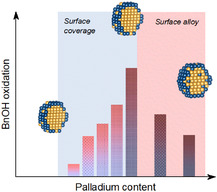

## 1 Introduction

The nonmonotonic increase in catalytic activity obtained by mixing two metals has intrigued catalysis scientists for years.^[^ ^1^, ^2^, ^3^ ^]^ Au is miscible with Pd in all the compositions, which facilitates obtaining AuPd alloys that are more reactive and stable than the corresponding monometallic catalysts for the catalytic oxidation of alcohols^[^ ^4^ ^]^ and direct synthesis of H_2_O_2_.^[^ ^5^, ^6^, ^7^ ^]^ The synergy between the two metals can be understood in terms of geometrical or ensemble effects, that is, one component may serve as a spacer to isolate the second metal surface atoms;^[^ ^8^, ^9^, ^10^ ^]^ or electronic or ligand effect, that is, charge transfer between the two metals;^[^ ^4^, ^11^ ^]^ or strain, that is, altering the width and center of the d‐band with regard to the Fermi level energy.^[^ ^12^ ^]^ Moreover, by changing the atomic ratio between the two metals, different atomic surface composition and configuration can be achieved, which may lead to a distinct composition‐dependent catalytic performance in a synergic or antisynergic fashion.^[^ ^1^, ^13^ ^]^ In addition to the different coordination environments experienced by a specific element within the nanoparticle, which is a geometric effect experienced when the atoms are located at low coordination sites versus at high coordination sites, quantum size effects, contingent upon the particle size, will play a role in changing the electronic configuration of the nanoparticle. Therefore, the delicate balance between these effects contributes significantly to the dynamics of chemical reactions and, ultimately, to achieving optimal performance. Even with all the advances in surface characterization and in situ or operando experimentation, controlling the most beneficial surface configuration in real catalysts remains a challenging task.

The surface composition in homogeneous bulk alloys is easily predicted,^[^ ^14^ ^]^ but bimetallic nanoparticle catalysts submitted to different chemical potentials, driven by temperature and gas atmosphere during the pretreatment and catalytic reaction processes, may experience surface segregation.^[^ ^15^ ^]^ De‐alloying of AuPd nanoparticles into Au‐rich core and Pd‐rich shell driven by heating and various atmospheres has been reported to further improve the benzyl alcohol oxidation turnover frequencies.^[^ ^16^, ^17^ ^]^ Such a surface configuration acts in equalizing the reactants and products binding energies closer to an optimal value. For instance, DFT calculations were used to explain the composition effect in reaching the volcano‐like activity of Au@Pd nanoparticles, prepared by the successive addition of Pd to preformed Au nanoparticles (NPs), in the oxidation of benzyl alcohol.^[^ ^3^ ^]^ The maximum activity was achieved at a Pd content of 10 mol% of Pd on top of the Au core of ≈12 nm, corresponding to about one monolayer of Pd. The key factor in increasing the catalytic activity derived from the balance between the number of active sites for substrate adsorption and the ease of product desorption, both of which are parameters extremely sensitive to Pd content.^[^ ^3^ ^]^ AuPd alloyed nanoparticles also presented the volcano‐shaped activity as a function of Pd content, but with maxima at an equimolar metal ratio (Au:Pd 1:1).^[^ ^1^ ^]^ In contrast, for the oxidation of glycerol using AuPd NPs, the maximum activity was achieved at a Au:Pd ratio of around 9:1^[^ ^18^, ^19^ ^]^ or even in single‐atom alloy systems, in which the amount of Pd is low enough so that Pd stays isolated, having only Au neighboring atoms.^[^ ^19^, ^20^ ^]^ This activity profile was not exclusive for oxidation: a volcano plot with maximum activity at a Pd content of ≈60% was reported for the hydrogenation of allyl alcohol catalyzed by AuPd alloyed NPs.^[^ ^21^ ^]^ More recently, Hutchings and collaborators^[^ ^22^ ^]^ opened a new avenue of possibilities for the development of bimetallic catalysts by showing that separating gold and palladium into isolated sites on the catalyst surface enhanced the catalytic activity for alcohol oxidation reactions. Likewise, we have recently reported similar effects when imposing the separation of Au and Pd through thermal treatments.^[^ ^16^ ^]^ These findings provide new guidance for designing bimetallic catalysts and new reaction approaches.^[^ ^23^, ^24^ ^]^

Herein, we present a systematic study aiming to investigate the structure–activity relationship of Au@Pd core‐shell NPs considering different Au core sizes and various amounts of Pd in the oxidation of benzyl alcohol (BnOH), and ultimately fine‐tuning activity of bimetallic AuPd catalysts. Au NPs of three different average sizes (2.6, 6.4, and 10.6 nm diameter) were examined, showing a volcano‐shaped activity with the maximum at different Pd at% for each of the Au core sizes tested, providing a proof‐of‐concept for the design of bimetallic catalysts. Moreover, by using X‐ray absorption spectroscopy (XAS) we were able to shed light on the turning point of the volcano‐shaped activity curve and to link the decrease of activity that occurs in Pd‐rich NPs (≈2 nm Au core) with AuPd alloying and surface restructuring.

## 2 Results and Discussion

### 2.1 Synthesis of Au@Pd Core‐Shell Catalysts and Catalytic Activity in the BnOH Oxidation

Colloidal Au NP seeds with varying average diameters (2.6 ± 0.4, 6.4 ± 0.9 and 10.6 ± 0.6 nm) were prepared following a literature method in which the different average particle sizes were only dependent on the synthesis temperature that is set as 40, 20 and 0 °C, respectively.^[^ ^25^ ^]^ UV‐vis absorption spectra of the Au NPs dispersed in toluene exhibit the characteristic Au surface plasmon absorption band at near 530 nm (Figure S1, Supporting Information). The band intensity increased and its position slightly red‐shifted as the nanoparticle size increases. The preformed Au NPs were thus immobilized on TiO_2_ with Au contents of 6.5 wt%, 5. 7 wt%, and 5.4 wt% Au, for 2.6, 6.4, and 10.6 nm, respectively. Particle sizes were estimated from TEM images of Au NPs in colloidal dispersion and of Au/TiO_2_‐supported catalysts (Figure S2, Supporting Information). Silica was also tested as a support, but titania was selected because it was able to immobilize the Au NPs without leaching during the purification steps and catalytic tests. Using a seed‐mediated approach, the stepwise preparation of core‐shell Au@Pd nanoparticles was performed by adding known volumes of a Pd(OAc)_2_ solution to Au/TiO_2_ (Au NPs seeds) followed by a reduction step with molecular hydrogen (4 bar H_2_, 100 °C, 30 min, Fisher‐Porter type reactor, in benzyl alcohol as solvent).

**Figure 1:**
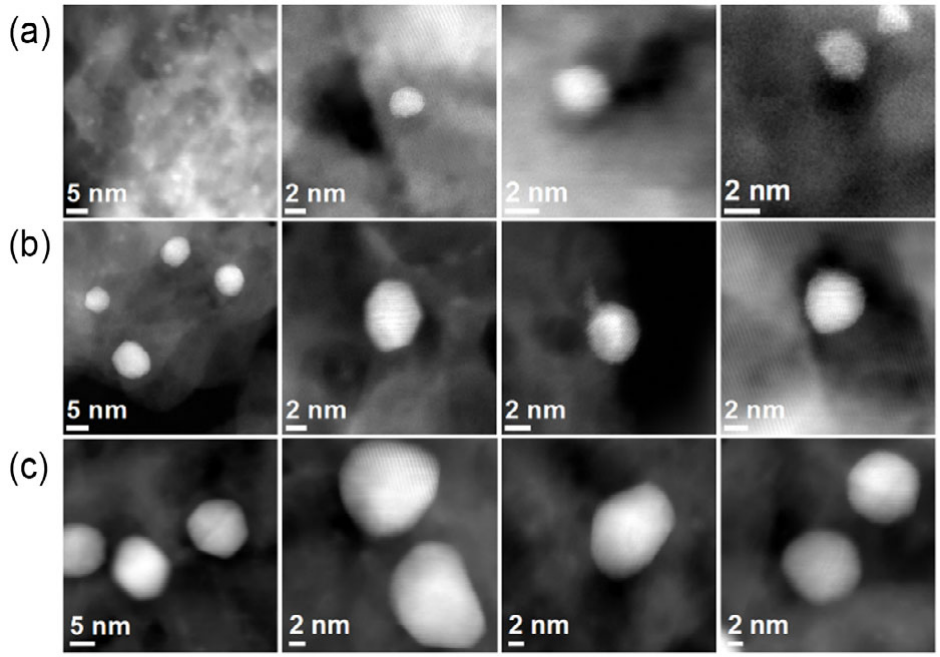
Representative STEM‐HAADF images of supported Au@Pd/TiO_2_ catalysts synthesized from Au core NPs of a) 2.6 nm, b) 6.4 nm, and c) 10.6 nm.

**Figure 2:**
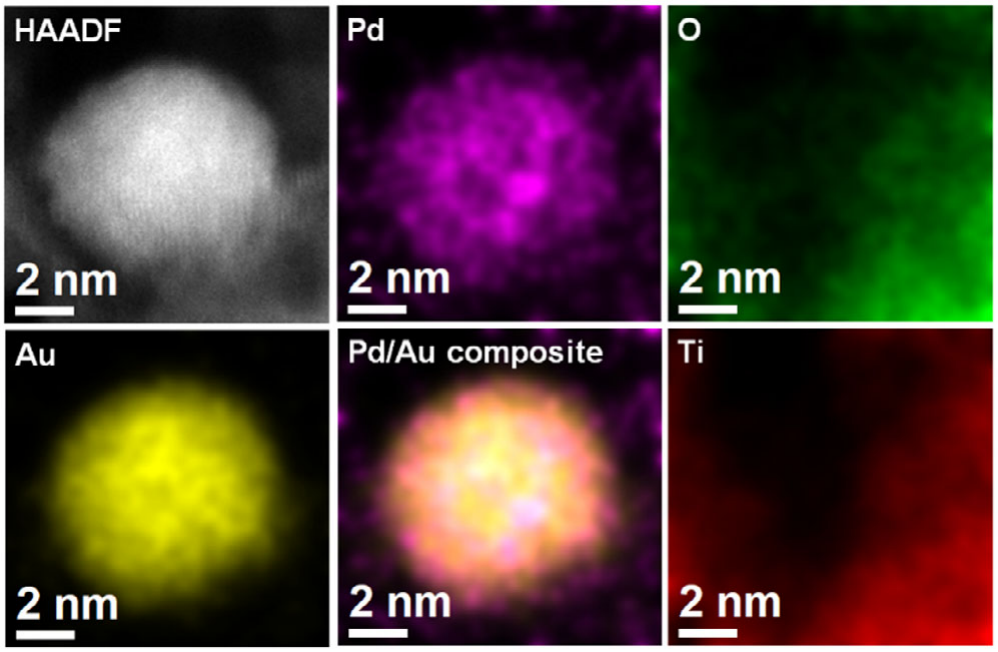
Representative STEM‐HAADF image of Au@Pd/TiO_2_ catalysts with Pd content of 40 at% on Au core of ≈9.3 nm (taken from sample with average 10.6 nm). Elemental EDS mapping of Au, Pd, and color mix of the two elements acquired for this NP.

Representative HAADF‐STEM images of the Au@Pd/TiO_2_ catalysts for the three different Au core sizes are shown in **Figure** **1**. HAADF‐STEM image and elemental mapping of Au@Pd nanoparticles (≈10 nm core, 40% Pd) are shown in **Figure** **2**. Au and Pd were homogeneously distributed in the particles of the zoomed region, while Pd is located on the Au surface. It is worth noting that isolated Pd nanoparticles on TiO_2_ were not found by HAADF‐STEM characterization.

**Figure 3:**
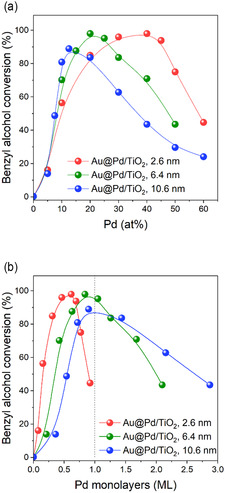
a) Conversion of benzyl alcohol using Au@Pd/TiO_2_ catalysts as function of the amount of Pd (at%) added to Au seeds of different average particle sizes. b) The same data points are converted in Pd monolayers. Reaction conditions: mol benzyl alcohol/mol_(Au+Pd)_ equal to 2500, P_O2_ of 6 bar at 100 °C, 150 min.

Three series of TiO_2_‐supported Au@Pd NP catalysts were obtained, one for each Au NP seed size, and with Pd content ranging from 5 at% up to 60 at% Pd (at% Pd is determined here as mol Pd/mol Au × 100), which correspond to diluted Pd atoms on top of Au seed NPs up to values close to one monolayer of Pd on the surface of Au seed NPs (see SI for further details and Table S1, Supporting Information). The Au@Pd/TiO_2_ samples were prepared in situ, in the same glass reactor used for the catalytic test. These synthesized catalysts were used in the oxidation of benzyl alcohol to benzaldehyde as a prototypical oxidation reaction. The reactor was first purged with N_2_, then loaded with molecular oxygen, and submitted to the desired reaction conditions and time (6 bar O_2_ and 100 °C). The results of conversion at a fixed time (2.5 h) as a function of the Pd molar fraction (at% Pd/Au) are summarized in **Figure** **3**a. The conversion of benzyl alcohol observed using the monometallic Au/TiO_2_ (blank experiment with 0 at% Pd) was close to zero, since gold requires the addition of a base to promote this reaction.^[^ ^26^ ^]^ The incremental addition of Pd to produce the Au@Pd/TiO_2_ catalysts resulted in a sharp increase in benzyl alcohol conversion for all the catalysts series. The maximum conversion was reached at specific Pd contents that were dependent on the average size of Au NPs seeds; further additions of Pd resulted in a sharp decrease in benzyl alcohol conversion (Figure 3a). Thus, the benzyl alcohol conversion as function of Pd added to Au follows a volcano shaped activity curve, while each Au core size required a different amount of Pd to reach the maximum activity: the 2.6 nm Au NP seed required ≈40 at% Pd and the 6.4 nm Au NP seed required ≈20 at% Pd while the 10.6 nm Au NP seed required ≈12.5 at% Pd. It is worth mentioning that the main product detected in all catalytic reactions is benzaldehyde, and benzoic acid was the only observed side product, as expected for gold and gold‐palladium catalysts. We could not find a correlation between the catalyst's structure and/or com.position and selectivity, which in all cases range from 70 to 100% (Table S2–S4, Supporting Information).

### 2.2 Particle Size and Composition Relationships in Determining Au@Pd Core‐Shell Structure

The amount of palladium added to prepare each Au@Pd/TiO_2_ catalyst shown in Figure 3a was converted to monolayers of Pd using the icosahedral full‐shell cluster model as the possible packing configuration of the Au NPs (see SI for details), and the data are shown in Figure 3b. The maximum activity values were achieved before the one monolayer of Pd threshold (Table S1, Supporting Information). As the size of the Au NPs increases, the at% Pd necessary to reach the maximum catalytic activity decreases, which agrees with the expected decrease in the ratio between the number of surface atoms per number of core atoms with size. The ratio between the atoms of Pd in the outermost full atom layer per atoms of Au in the core (total) is defined here as the at% Pd. The amount of Pd in the final Au@Pd/TiO_2_ catalysts that showed the best catalytic activity is very close to the estimated Pd monolayer values for the samples having Au NPs of 6.4 and 10.6 nm. Nevertheless, there is a significant difference between the at% Pd for maximum activity and the at% Pd to reach a Pd monolayer for the sample comprised of 2.6 nm Au NPs, in which the experimental Pd content to achieve maximum activity is close to 0.5 monolayer. This difference in experimental Pd content to the estimated Pd content to achieve a monolayer is beyond the possible experimental errors, such as those inherent in weighing both Au and Pd precursors and TiO_2_, or measuring volume of the palladium solution, or even variations in temperature and pressure of the reactional conditions, which may have contributed to eventual deviations on the calculated values.

**Figure 4:**
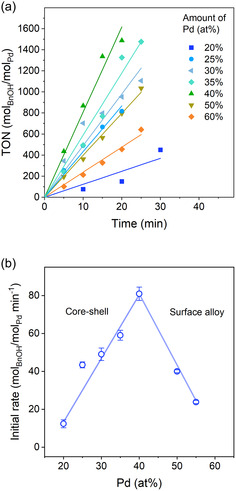
a) Kinetic curves for benzyl alcohol oxidation catalyzed by Au@Pd/TiO_2_, with Au NPs core of 2.6 nm. b) The initial reaction rate derived from the kinetic curves in (a) and are plotted as function of Pd content in the Au@Pd system. Reaction conditions: molar ratio (mol_BnOH_/mol_(Au+Pd)_) equal to 2500, 6 bar of O_2_ and 100 °C.

To investigate the behavior of the 2.6 nm Au seeds in more detail, new catalytic tests were carried out to follow the evolution of the reaction with time for Au@Pd/TiO_2_ catalysts prepared with different amounts of Pd (**Figure** **4**a). Volcano curves for activity in Pd‐based catalysts as function of particle sizes have been reported in the literature, with the assumption that the ratio between high‐coordination sites and low‐coordination sites modulated by increasing particle sizes, result in the loss of activity for benzyl alcohol oxidation, whereas at small NPs, the electron density shifts toward the Fermi level,^[^ ^12^, ^14^ ^]^ and therefore, the binding strength between the adsorbed species to the Pd atoms are higher, which also cause a decrease in the overall activity. In this case, the maximum activity is governed by weakening the electronic size effects, while having a higher amount of low‐coordinated Pd sites, which have been found to be around 4 nm in cuboctahedra Pd NPs. At a monolayer level, such assumption can explain why the maximum activity is found to be at Pd atom coverages close to the monolayer in the case of the Au cores of ≈6 nm and ≈10 nm. It has been shown that finite size effects change dramatically the binding energies for CO and O_2_ on Au nanoparticles smaller than 2.6 nm, and above this limit, the surface of the nanoparticles behaves like an extended surface of bulk materials.^[^ ^10^ ^]^ Therefore, without the finite size effects of the Au core NPs, the surface of these nanoparticles can act as substrate for the overlaying Pd atoms, which will populate more the flat Au surfaces. The conversion curves presented in Figure 3 show that the maximum activities were centered at similar Pd coverages for the 6.4 and 10.6 nm Au NPs (close to the amount required for a full monolayer), agreeing with the previous theoretical and experimental studies of Au and Pd NPs. Based on that, and on the results of Figure 3, we have considered that each addition of Pd up to a monolayer on top of Au can be considered as an active site. Therefore, the turnover numbers (TON) of Figure 4a correspond to mol of benzyl alcohol converted per mol of Pd. The initial reaction rate (r_0_), in mol_BnOH_/mol_Pd_ min^−1^, for each different amount of added Pd, is shown in Figure 4b. The r_0_ versus Pd at% profile shows maximum activity at 40 at% Pd, an amount that corresponds to 0.57 monolayer. It is worth mentioning that a Pd monolayer would require 65 at% Pd for Au NPs of 2.6 nm. Accordingly, the obtained kinetic data confirm the volcano‐like behavior for the synthesized Au@Pd/TiO_2_ catalysts, as observed in previous experiments without kinetic tracking (Figure 3). Therefore, there is an optimal percentage of palladium needed in the bimetallic system to achieve the best catalytic activity, which is below a palladium monolayer.

### 2.3 XAS Study of Au@Pd Core‐Shell Structure

**Figure 5:**
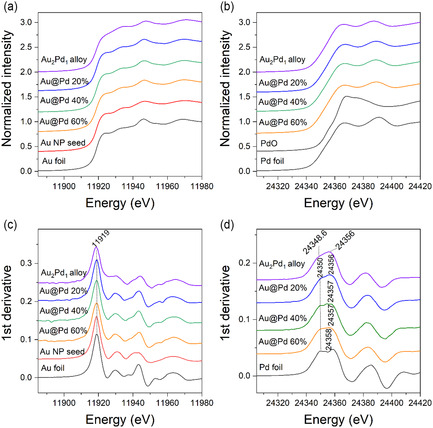
XANES spectra at the a) Au L_3_‐edge and b) Pd K‐edge obtained for the c,d) Au@Pd/TiO_2_ catalysts, with their respective 1st derivative.

The local structure of the series of catalysts prepared with average 2.6 nm diameter Au seeds after addition of 20 at%, 40 at%, and 60 at% Pd was analyzed by X‐ray absorption spectroscopy (XAS). The spectra were acquired at room temperature, exposed to air, at Au L_3_‐edge and Pd K‐edge. The oxidation state was evaluated by comparison with metal foils of Au and Pd and PdO references. The XAS of a reference sample of AuPd homogeneous alloy nanoparticles (≈3 nm average size) with a stoichiometry 2:1 (represented as Au_2_Pd_1_) was measured. This sample was prepared in our laboratory, as reported in a recent work.^[^ ^16^ ^]^ **Figure** **5** shows the X‐ray absorption near‐edge spectra (XANES) obtained for the samples and reference compounds; in the lower panel, the first derivatives of the top panel spectra. The peak at 11919.0 eV (Figure 5c) corresponds to the Au L_3_‐edge energy while the peak at 24350.0 eV (Figure 5d) corresponds to Pd K‐edge, thus indicating that both elements were predominantly in metallic state in the Au@Pd/TiO_2_ catalysts.

**Figure 6:**
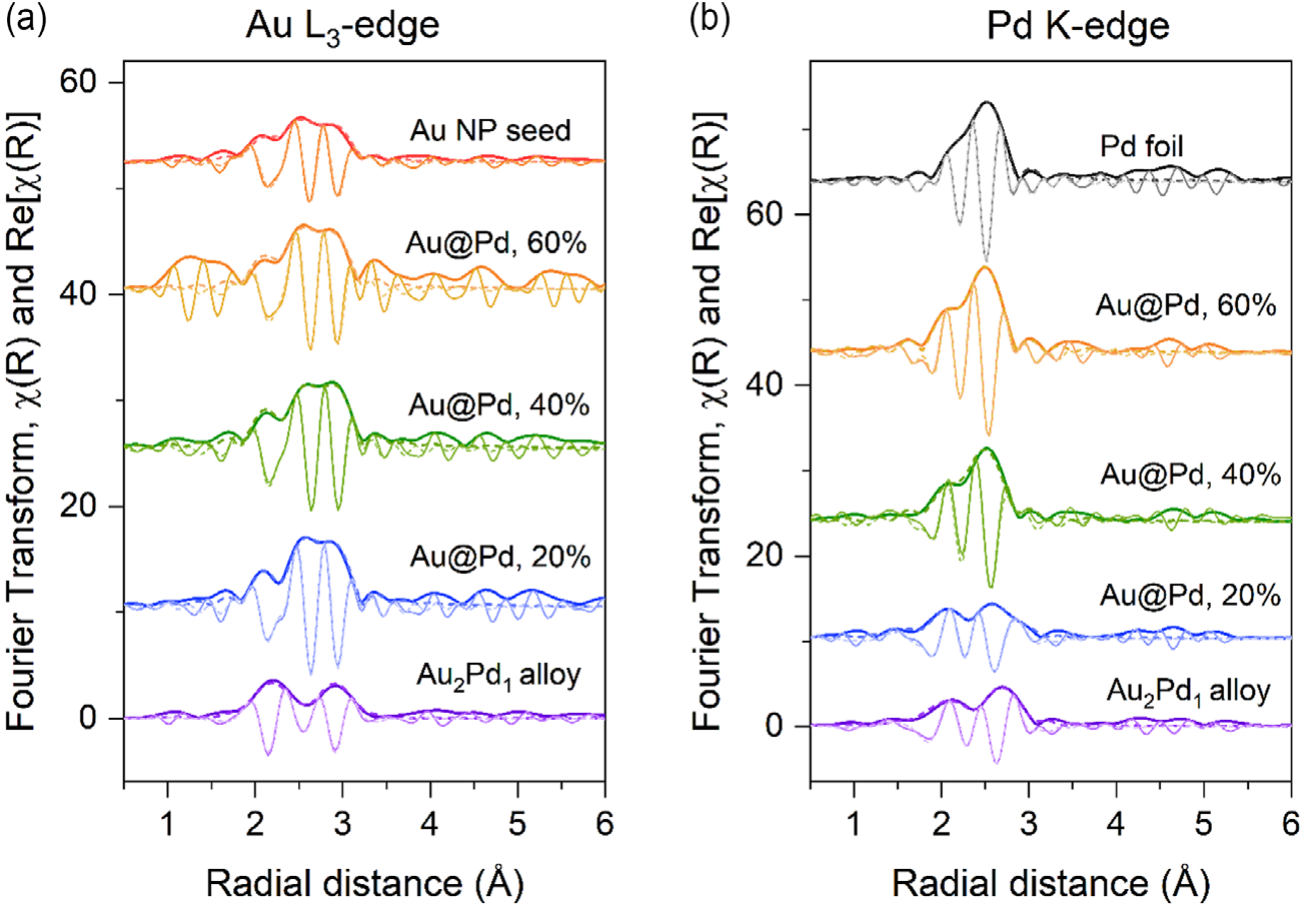
k^3^‐weighted real part and magnitude of Fourier transform XAS spectra (solid lines) and respective best fit (dash lines) at the a) Au L_3_‐edge and b) Pd K‐edge obtained for the Au@Pd/TiO_2_ catalysts.

**Figure** **6** shows the real part and magnitude of the Fourier transform of the XAS spectra for the catalysts investigated. The spectra in k‐space are shown in Figure S3, Supporting Information. The Au L_3_‐edge spectra for the Au@Pd samples are similar to the ones of monometallic Au NPs. At Pd K‐edge, the sample with 20 at% Pd shows an EXAFS spectrum like that of the Au_2_Pd_1_ alloy NP. Thus, at this concentration, Pd atoms interact more with Au atoms than with Pd atoms, that is, Pd is well dispersed on the surface of gold. However, as the Pd concentration increases, the spectra begin to resemble that of bulk‐like Pd (Pd foil reference in Figure 6). This suggests that there are more Pd neighbors to Pd, as would be expected in a core‐shell configuration, and that Pd is forming clusters or aggregates on top of gold.

**Figure 7:**
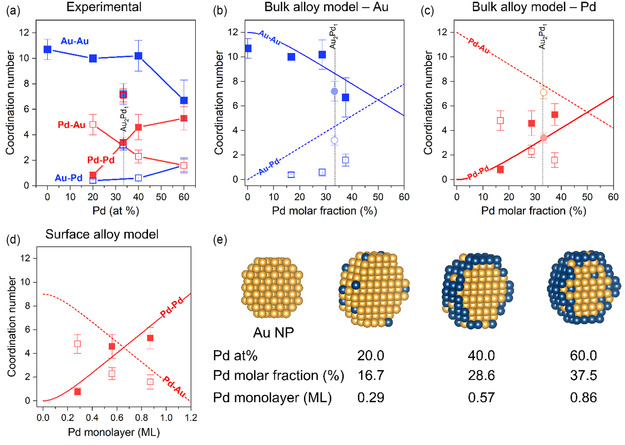
a) Evolution of the coordination number for Au@Pd/TiO_2_ catalysts, as a function of the amount of Pd on top of Au nanoparticles (2.6 nm). b) The CNs of Au—Au and Au—Pd are plotted considering a bulk alloy model (with Pd molar fraction) and Pd—Pd and Pd—Au are shown in (c). CNs for Au_2_Pd_1_ alloy nanoparticles are also shown as a reference. d) The same data points are plotted considering a surface alloy model (with Pd content being the number of Pd atoms respective to the number of Au atoms present in the Au NPs presynthesized). The full and dash lines represent the estimated CN values from the bulk and surface alloy models, as function of Pd content. e) Scheme of the evolution of core‐shell configuration with increasing Pd content.

The best‐fit EXAFS models included all combinations of Au—Au, Au—Pd, Pd—Pd, and Pd—Au scattering paths for the different samples. We found important variations in the coordination numbers (CN) as a function of the amount of Pd present in the sample. Table S4, Supporting Information, shows the parameters obtained from the best‐fit models and **Figure** **7**a shows the coordination numbers as a function of the Pd content. The coordination number for the Au—Au bond (CN_Au‐Au_) is around 10.7 ± 0.8 for pure Au NPs on TiO_2_. This value is lower than the CN_Au‐Au_ of Au bulk (CN_Au‐Au bulk_ = 12) but is expected for nanoparticles in the range of 2 to 3 nm.^[^ ^27^ ^]^ The addition of 20 and 40 at% Pd does not significantly change the CN_Au‐Au_. The sample with 60 at% Pd showed a lower CN_Au‐Au_ of ≈6.7±1.6. The values obtained for Au—Pd scattering (CN_Au‐Pd_) are close to 0.5 for the 20 at% and 40 at% Pd samples, and again different for the 60 at% Pd sample, which presented a CN_Au‐Pd_ of 2.3 ± 0.7. On the other hand, the values for Pd—Pd scattering (CN_Pd‐Pd_) undergo an increase with the addition of Pd from CN_Pd‐Pd_ of 0.8 ± 0.3 (20 at% Pd) to 4.8 ± 0.8 (40 at% Pd), and then to 5.3 ± 0.9 (60 at% Pd). Concomitantly, the Pd—Au scattering (CN_Pd‐Au_) decreases in an opposite trend from CN_Pd‐Au_ of 4.8 ± 0.8 (20 at% Pd) to 2.3 ± 0.5 (40 at% Pd) and then 1.6 ± 0.5 (60 at% Pd).

We have used two theoretical models to estimate the evolution of CNs as function of Pd content: a bulk alloy model (Figure 7b,c) and a surface alloy model (Figure 7d), as proposed by Boubnov et al.^[^ ^28^ ^]^ In the case of surface alloy model, the CN values are plotted against the Pd monolayer values, which is a more intuitive way of representing the Pd coverage on top of a Au core. Comparing the CN experimental values with the ones estimated for a bulk Au—Pd alloy, it is clear that Au‐Pd contribution does not approach the expected values for a bulk alloy, and that Au—Au remains almost unchanged until high Pd content (60 at%) is reached. In the case of CN_Pd‐Pd_ and CN_Pd‐Au_, the discrepancy between experiment and model is even greater. Unlike that, the CN values obtained for the Au_2_Pd_1_ reference sample are very close to the estimated values for the bulk alloy model. Thus, a bulk alloy system does not represent well the bimetallic Au‐Pd structure reached here. However, if the model considers the surface termination, the experimental CN_Pd–Pd_ values agree well with the calculated ones. Regarding the CN_Pd–Au_ values, the experimental values are still lower than the expected ones; however, they are much closer to the surface alloy model than the bulk alloy model.

The results of EXAFS suggests that for Au@Pd/TiO_2_ catalysts with Pd/Au = 20 and 40 at%, palladium is deposited on gold (increase in Pd‐Pd CN), but with Pd/Au = 60 at%, palladium seems to mix with the gold forming an alloy, suggesting that this may be the reason for the deactivation of the catalyst and not necessarily the formation of a palladium bilayer, as suggested for larger nanoparticles.^[^ ^3^ ^]^ With Pd dissolution to the core of the nanoparticles, the number of Pd atoms on the surface should decrease, causing a decrease in active sites. Besides that, the formation of a surface alloy can change the lattice constants and the electron density of the parent elements, corresponding to a combination of ensemble and ligand effects, which determine the adsorption properties of the catalytic surface. The Au@Pd/TiO_2_ catalysts with Pd/Au = 40 at% provide the most favorable configuration for the reaction steps involved in benzyl alcohol oxidation.

## 3 Conclusion

It was already well known that bimetallic gold‐palladium catalysts outperform their monometallic counterparts for oxidation of benzyl alcohol model reaction; herein, we found that core‐shell type catalysts are more active than alloyed AuPd. The proportions of gold and palladium required to achieve the formation of core‐shell structures and consequently the maximum catalytic activity depends both on the size of the core of gold and the amount of palladium added. For gold nanoparticles larger than 6 nm, the addition of Pd increases activity and the amount of palladium needed to obtain maximum activity can be estimated to correspond approximately to a palladium‐on‐gold monolayer. Smaller nanoparticles (2–3 nm) cannot be ruled by this model and maximum activity was obtained by adding less amounts of palladium than predicted for the Pd monolayer; the maximum activity was reached at 0.5 monolayer Pd. Our investigation showed that structural evolution (alloying) decreased the activity of the bimetallic nanoparticles, thus creating core‐shell architectures, where gold is in the core and palladium in the shell, which is key to enhancing the catalytic performance. The insights gained here suggest that the rational design of bimetallic catalysts should involve a systematic study of catalyst structure (core‐shell/alloy), particle size, and composition. We are currently investigating the effect of core‐shell and alloyed AuPd catalysts on hydrogenation reactions.

## 4 Experimental Section

All the reagents used were purchased and used as received. TiO_2_ (Sigma–Aldrich, nanopowder < 25%, anatase, surface area 45–55 m^2^ g^−1^, 99.7% trace metal basis) was used as support; HAuCl_4_ solution (99.99% Au), 30 wt% in dilute HCl; Pd(OAc)_2_ (98 %); 1,2,3,4‐tetrahydronaphthalene (tetralin, anhydrous, 99%) and tert‐butylamine borane complex (TBAB, 97%) were purchased from Sigma–Aldrich. Oleylamine (80–90%) was purchased from Acros Organics. Acetone was purchased from Labsynth. Gases (O_2_, N_2_, H_2_, Ar, and synthetic Air, all grade 5.0) were purchased from Gama Gases. The glass reactors were thoroughly cleaned with aqua regia solution (HCl:HNO_3_ = 3:1 v/v), rinsed with copious deionized water, and then dried in an oven prior to use.

4.1

4.1.1

##### Au/TiO_2_ Catalyst Preparation

The TiO_2_‐supported Au NP catalysts were obtained in a two‐step sol immobilization method. Au NPs colloids of different average sizes were prepared using a procedure reported by Peng et al.^[^ ^25^ ^]^ In this procedure, the only parameter to tune the average nanoparticle size is the synthesis temperature; herein, temperatures of 40, 20, and 0 °C were used to produce Au NPs of ≈2, 6, and 10 nm, respectively. Typically, a solution containing 10 mL tetralin, 287 mg (0.254 mmol) HAuCl_4_, and 10 mL of oleylamine was kept in an ice bath at 0 °C. After 10 min, a reducing solution containing 44 mg (0.5 mmol) TBAB, 1.0 mL tetralin, and 1.0 mL oleylamine was added to the former solution. The orange‐colored solution changed to a brownish‐red color after the addition of the reducing solution. The solution (Au sol) was kept under stirring at 0 °C for 1 h and then the ice bath was removed to let the solution reach room temperature. This procedure was repeated at different temperatures (20 and 40 °C) to produce Au sols with smaller particle sizes. The increase in temperature leads to a decrease in the size of the nanoparticles due to the increase in the solubility of the precursor in the oleylamine/tetralin mixture. Furthermore, at higher temperatures, the gold reduction process is faster, increasing the number of nucleation centers, and thus, smaller particles are produced. After the Au sol reached room temperature, 100 mL of acetone and 1.0 g of the TiO_2_ support were added and the mixture was kept under stirring overnight. The solid was recovered by centrifugation, washed 5 times with acetone within repeated centrifugation‐resuspension steps, and dried under vacuum.

##### Au@Pd/TiO_2_ Catalyst Preparation

Au@Pd/TiO_2_ bimetallic catalysts were prepared by adding different amounts of palladium (in at%) corresponding to the gold present in the Au/TiO_2_ catalyst material containing Au NPs of ≈2, 6, and 10 nm. The preparation consisted of adding a desired amount of a freshly prepared Pd(OAc)_2_ solution in benzyl alcohol ([Pd^2+^] = 1.68 × 10^−3^ mol L^−1^) to 50 mg of Au/TiO_2_ catalyst loaded in a Fischer–Porter 100 mL glass reactor. Then, more benzyl alcohol was added to achieve the desired substrate‐to‐metal ratio (2500 mol/mol). The reactor is equipped with a magnetic bar for stirring, a ball valve to allow reaction sample collection, and a needle valve to allow the admission of gas. The reaction mixture was kept under magnetic stirring and the reactor was purged and pressurized with H_2_ at 4 bar and heated to 100 °C, immersed in an oil bath, for 30 min. Following, the reactor was cooled to room temperature, and hydrogen gas was released from the reactor with subsequent purge with N_2_; following, O_2_ was added to the reactor vessel for the catalytic experiments. The catalysts were labeled as Au@Pd x%, where *x* is the percent ratio of Pd added regarding Au, in mol basis (mol Pd/mol Au × 100).

##### Au_2_Pd_1_ Alloy Reference Catalyst Preparation

AuPd bimetallic alloy catalysts were prepared following a procedure described in a previous work.^[^ ^16^ ^]^ Briefly, defined volumes of aqueous solutions of HAuCl_4_ ([Au^3+^] = 4.85×10^−3^ mol L^−1^), K_2_PdCl_6_ ([Pd^2+^]= 2.25×10^−3^ mol L^−1^) and polyvinyl alcohol (PVA) (1% m/V) were added to a synthesis flask and completed with deionized water (50 mL), to produce nanoparticles of Au/Pd atomic ratio of 2; the solution was magnetically stirred at room temperature for 30 min, when the reducing agent was add to the flask, NaBH_4_ (0.1 mol L^−1^). The color of solution changed instantaneously from dark yellow to purple, indicating reduction of the metals. The dispersion was kept under stirring for another 30 min, when SiO_2_ (synthesized according to ref. 16) was added to the flask (amount enough to produce 1 wt% Au_2_Pd_1_ NPs on SiO_2_), serving as a support carrier for the nanoparticles. The solid material was recovered by centrifugation and washed twice with 25 mL of warm deionized water (60 °C) and one time with 25 mL of ethanol. Finally, the solid was allowed to dry at room temperature. This procedure generated Au_2_Pd_1_ NPs of 2.5 ± 1.2 nm supported on SiO_2_ (1 wt% Au + Pd).

##### Catalytic Evaluation

Each Au@Pd/TiO_2_ catalyst was prepared immediately before use. The catalytic tests were performed in the same reactor, without isolating the catalyst. Typically, the Fischer–Porter 100 mL glass reactor containing each of the Au@Pd/TiO_2_ catalysts in benzyl alcohol, prepared as above to contain × at% Pd, was purged three times with O_2_, leaving the vessel at 6 bar. Similar experiments were performed with Au/TiO_2_ catalysts without palladium, corresponding to a blank experiment with 0 at% Pd. Typically, the temperature was maintained at 100 °C with an oil bath on a hotplate stirrer connected to a digital controller (C‐MAG HS, IKA). During the reaction, the reactor is connected to an O_2_ gas manifold, ensuring a constant pressure of O_2_ at 6 bar. For the reactions performed at a fixed reaction time, after 2.5 h, the reactor was cooled down to room temperature, depressurized; the products were collected with a glass syringe, isolated by centrifugation, and diluted in cyclohexane for analysis by gas chromatography (GC). To obtain kinetic curves, the reactions were performed in the same manner, however, sampling at various time points. The GC analysis was performed in a Shimadzu GC‐2010 instrument, equipped with a RTx‐Wax capillary column (30 m × 0.25 mm × 0.25 mm) and a flame ionization detector (CG‐FID). Conversions and selectivity were calculated based on the peak areas of the chromatograph, calibrated with biphenyl as an internal standard.

##### Characterization Methods

TEM analyses were performed using a JEOL 2100 instrument. Catalyst samples for TEM were prepared by sonicating the colloidal dispersion of Au NPs or the catalyst powder in propan‐2‐ol. A drop of the resulting dispersion was placed on a carbon‐coated copper grid (Ted Pella). The histogram of nanoparticle size distribution was obtained from the measurement of about 200 particles. The HAADF‐STEM images were acquired using a Thermo Scientific Titan Cubed Themis equipment, with acceleration voltage of 300 kV and double aberration corrected; compositional EDS mapping was performed with a Bruker Espirit system of four windowless EDS detectors. The quantification of Au and Pd in the catalytic support was performed using the FAAS technique, performed in a Shimadzu AA‐6300 atomic absorption spectrometer; the powder samples (15 mg) were treated with 8 mL of aqua regia under heating for 2 h. After cooling, the final solutions were diluted to 25 mL, centrifuged, and the liquid part was analyzed. The UV‐Vis spectra, collected in a Shimadzu UV‐1700 spectrometer, were obtained for the colloidal Au sol, dispersed in hexane, using in a quartz cuvette with an optical path of 1 cm.

XAS spectra on Au L_3_‐edge and Pd K‐edge were measured on the 2‐2 beamline of Stanford Synchrotron Radiation Laboratory (SSRL), a bending magnet beamline equipped with a double crystal monochromator (Si (220), φ = 90°). The beam size was ≈5 mm horizontal and 1 mm vertical. The acquisition of X‐ray absorption spectra was done in fluorescence mode using a PIPS detector. Pd and Au foils were used as a reference to correct edge shifts and were measured simultaneously in transmission mode. The Au@Pd/TiO_2_ catalysts recovered after reaction (around 30 mg) in powder form were loaded to quartz capillaries (100 mm long, 3 mm O.D. and wall thickness of 0.2 mm) and accommodated between quartz plugs in the center of the capillary, giving a sample length of around 10 mm. Spectrum calibration and normalization were done using Athena software, and modeling of EXAFS spectra was performed using Artemis software, both from Demeter package.^[^ ^29^ ^]^ The scattering paths of Au—Au, Au—Pd, Pd—Pd, and Pd—Au were generated by FEFF8 code, considering crystallographic information from Au (hcp) and Pd (fcc) lattices.

## Conflict of Interest

The authors declare no conflict of interest.

## Supporting information

Supplementary Material

## Data Availability

The data that support the findings of this study are available from the corresponding author upon reasonable request.
