# Supplementary material for: Volcano‐like Activity Trends in Au@Pd Catalysts: The Role of Pd Loading and Nanoparticle Size
Source: Chemphyschem. 2025 Jul 3;26(16):e202500164. doi: 10.1002/cphc.202500164 (PMC12388158; doi:10.1002/cphc.202500164)
Supplement: Supplementary file 1 — Supplementary Material [file CPHC-26-e202500164-s001.pdf]

Supporting information

Volcano-like Activity Trends in Au@Pd Catalysts: The Role of Pd Loading and Nanoparticle Size

Adriano H. Braga,[1] Jhonatan L. Fiorio,[1] Ofelia Yang,[1] Karla L. Caetano,[1] Tiago Artur Silva,[1] Adam S. Hoffman,[2] Simon R. Bare,[2] Jefferson Bettini,[3,4] Naga Vishnu V. Mogili[3] and Liane M. Rossi[1]*

[1]*Departamento de Química Fundamental, Instituto de Química, Universidade de São Paulo, São Paulo 055008-000 (Brazil)*

[2]*Stanford Synchrotron Radiation Lightsource, SLAC National Accelerator Laboratory, Menlo Park, California 94025 (USA)*

[3]*Brazilian Nanotechnology National Laboratory (LNNano), Brazilian Center for Research in Energy and Materials (CNPEM), Campinas, São Paulo 13083-100 (Brazil)*

[4]*School of Technology, University of Campinas (UNICAMP), Limeira, São Paulo 13484-332 (Brazil)*

Model used to estimate the number of surface atoms

Nanoparticles are formed by a finite set of atoms grouped in polygonal and polyhedral geometries of high symmetry and high packing efficiency. The most stable clusters contain characteristic numbers of atoms, called "magic numbers".[1] These numbers follow a sequence that results from rule-guided packaging governed by electronic and steric principles. Magic numbers and sequences in cluster formation are linked to nucleation and growth processes, which are governed by the frequent competition between packaging factors and binding energies. As these processes can be kinetically or thermodynamically controlled and thus are sensitive to experimental conditions, the effects of binding force (electronic parameters) and packing parameters (steric effect) imply different magical sequences resulting from successive fillings of electronic and atomic layers. Considering the construction of these geometries from a single atom, the growth of a nanoparticle takes place from a basic structure that increases through the addition of other atoms. As the cluster gains a new atom, a rearrangement occurs until it forms a new layer of atoms that assumes a more stable structure, and so on. These stable structures follow the sequence of magic numbers and can be calculated through the following equations 2 and 3.

$$Gn = \frac{10}{3}n^3 + 5n^2 + \frac{11}{3}n + 1 \qquad n\geq 0 \qquad (2)$$

$$Sn = 10n^2 + 2 \qquad n\geq 1 \qquad (3)$$

Adopting the layer-by-layer growth model for icosahedral structures, from these equations, it is possible to determine the total number of atoms in a nanoparticle (Gn), distinguishing them from surface atoms (Sn) and from internal atoms (In) for each stable structure of the sequence. This information is very useful for calculating the

number of atoms on the surface of a nanoparticle from the composition and size information. In addition, it can be used to understand the core-shell structure that can be formed by the controlled combination of two metals. Here, it was used to estimate the amount of Pd that has to be added to the Au seeds to prepare bimetallic core-shell catalysts.

Taking the nanoparticle with an average diameter of 2.6 nm as an example, we consider the radius of 1.3 nm and thus the volume of a sphere ($V_{sphere} = 4/3\pi r^3$) with this radius is 9.2 nm^3. Knowing that gold atoms are packed in a FCC (face-centered cubic) structure, we consider the lattice parameters a = b = c = 0.4065 nm and determine that the volume occupied by a unit cell (V_{cell}) is 0.067 nm^3. Then, the volume of the sphere was divided by the volume of a unit cell to obtain the number of cells in the nanoparticle (N). Thus, N = 137 unit cells. As each unit cell consists of 4 gold atoms, the number of cells was multiplied by this value, resulting in a total number of atoms (Gn) of 548. To find the number of layers (n) present in a 2.6 nm nanoparticle, we apply equations 2 and 3 and determine that 548 atoms correspond to 5 layers of gold, which it contains, according to the full-layer cluster model, 561 atoms. Considering that in Au@Pd core-shell structures, Pd will be present in the next layer (n+1), it was possible to find the number of surface Pd atoms (Sn+1) necessary to cover the gold nanoparticle with an atomic layer of palladium. Thus, with n+1 = 6 layers, we calculate that Sn+1 = 362. From this, we determine the atom percentage (at%) of palladium needed to be added to the Au nanoparticle of 2.6 nm to obtain a Pd monolayer of Pd on the Au core is 64.5 at% of palladium in relation to the gold present in the gold core ((Sn+1/Gn)*100). The same calculation was repeated for the 6.4 nm and 10.6 nm nanoparticles, resulting in 23.9 at% and 13.9 at% palladium, respectively (table S1). In this way, the amount of palladium to be added to the gold was planned, considering the different sizes of the gold cores. All bimetallic preparations were made in situ, in the reactor itself and in the reaction medium used for the catalytic tests of oxidation of benzyl alcohol.

Table S1. Estimated amount of palladium atoms (at%) necessary to coat the Au core with a Pd monolayer ((Sn+1/Gn)*100).

**Au NP size (nm)**	**Pd monolayer (at%)**
2.6	64.5
6.4	23.9
10.6	13.9

Catalytic activity in the benzyl alcohol oxidation:

Table S2. Conversion of benzyl alcohol and benzaldehyde selectivity for 2.6 nm Au/TiO_2 and the corresponding Au@Pd/TiO_2 catalysts.

Pd (at %)	Conversion(%)	Selectivity(%)
0	0.2	100
5	16	87
10	56	74
20	85	71
30	96	88
**40**	**98**	**88**
45	94	89
50	75	88
60	45	96

Reaction conditions: mol benzyl alcohol/mol$_{(Au+Pd)}$ equal to 2500, P_{O2} of 6 bar at 100 °C, 150 min.

Table S3. Conversion of benzyl alcohol and benzaldehyde selectivity for 6.4 nm Au/TiO_2 and the corresponding Au@Pd/TiO_2 catalysts.

Pd (at %)	Conversion (%)	Selectivity (%)
0	0.2	100
5	14	93
10	70	89
15	88	89
**20**	**98**	**89**
25	95	86
30	84	87
40	71	88
50	44	85

Reaction conditions: mol benzyl alcohol/mol$_{(Au+Pd)}$ equal to 2500, P_{O2} of 6 bar at 100 °C, 150 min.

Table S4. Conversion of benzyl alcohol and benzaldehyde selectivity for 10.6 nm Au/TiO_2 and the corresponding $Au@Pd/TiO_2$ catalysts.

Pd (mol %)	Conversion (%)	Selectivity (%)
0	0.3	100
5	14	93
7.5	49	93
10	81	92
**12.5**	**89**	**89**
20	84	90
30	63	88
40	44	85
50	30	87
60	24	88

Reaction conditions: mol benzyl alcohol/$mol_{(Au+Pd)}$ equal to 2500, P_{O2} of 6 bar at 100 °C, 150 min.

Additional characterization

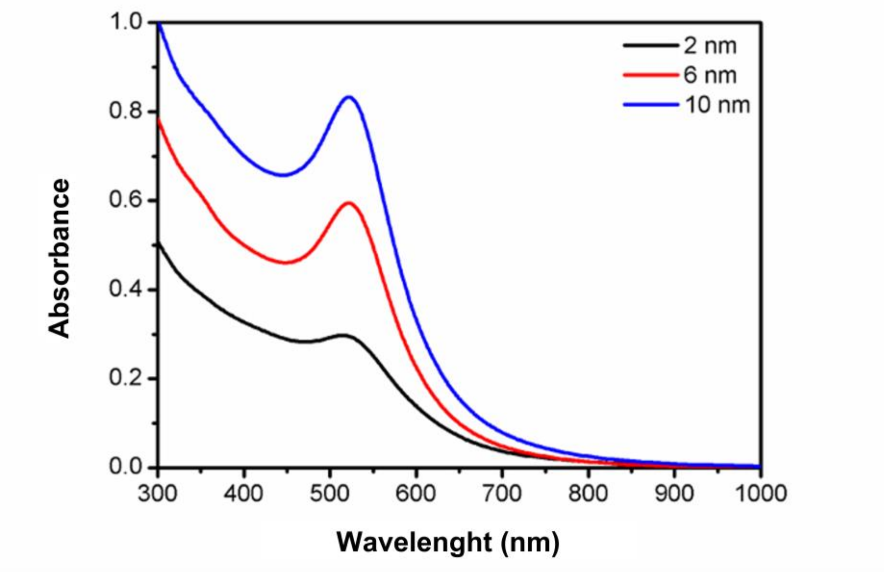

Figure S1. UV-Vis spectra of Au NPs synthesized at 40 °C, 20 °C and 0 °C.

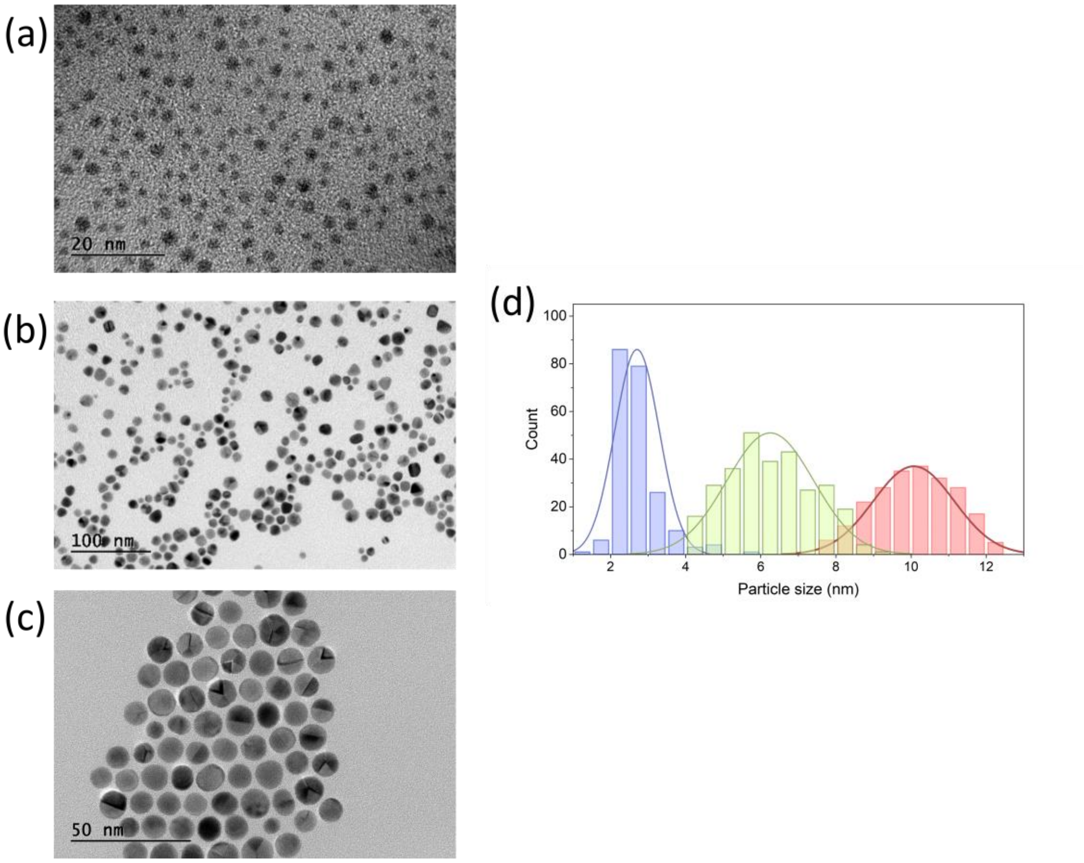

Figure S2: Representative STEM-HAADF images of supported Au NPs synthesized at (a) 40 °C (to yield ~2.6 nm), (b) 20 °C (to yield ~6.4 nm) and (c) 0 °C (to yield around 10.8 nm. Histograms of particle sizes distribution (d) obtained from the TEM images of the Au NPs; approximately 300 particles were counted for each sample.

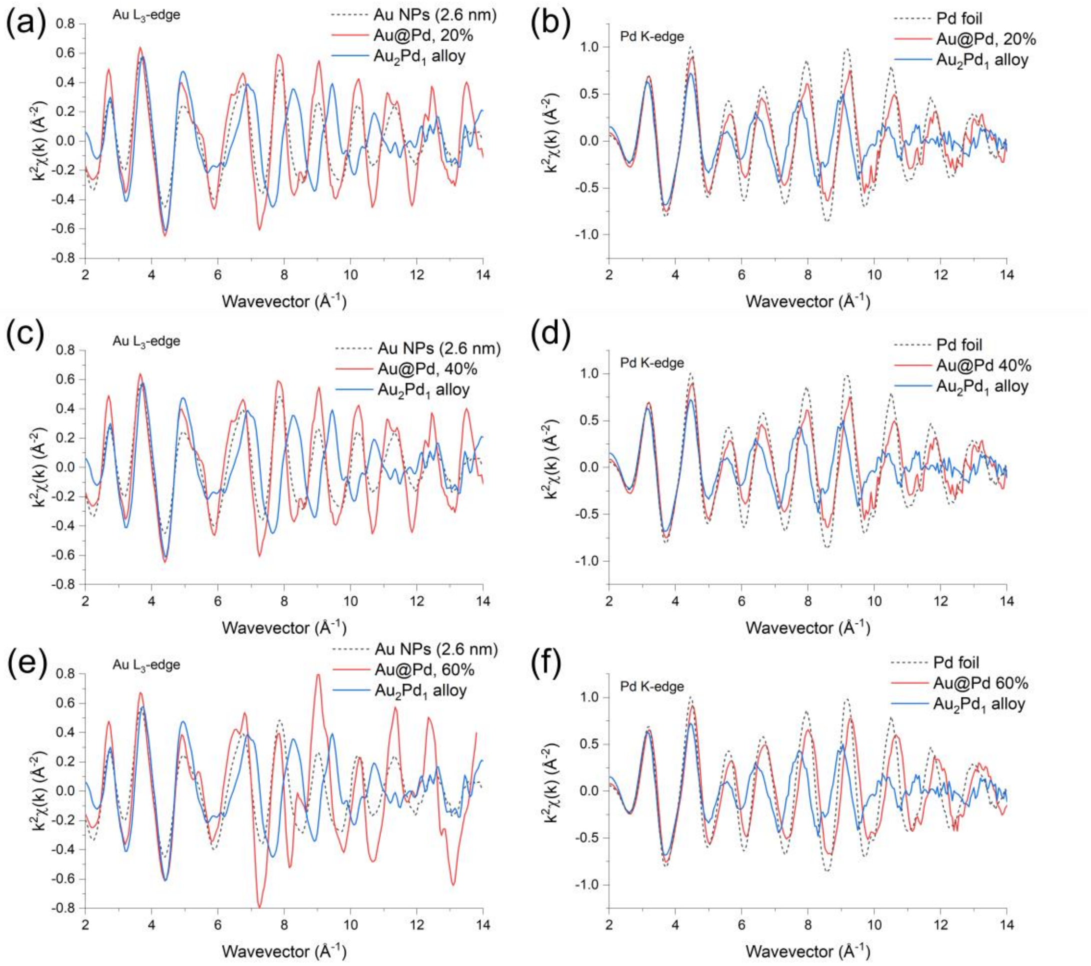

Figure S3: k^2-weighted EXAFS spectra of the Au@Pd/TiO_2 catalysts with 2.6 nm core Au NPs and varying amounts of Pd. (a-b) Au@Pd/TiO_2 with 20 at%, (c-d) 40 at% and (e-f) 60 at% of Pd on Au L_3-edge and Pd K-edges.

Table S5: Best fit parameters obtained from EXAFS data of Au@Pd/TiO_2 samples of 2.6 nm Au NPs.

Sample	E_0 (eV)	Scattering path	Coordination Number	Radial distance (Å)	Debye-Waller factor, σ^2 ($\times 10^{-3}$ Å^2)	χ^2_{red}
References						
Au foil	4.3±0.3	Au-Au	12	2.88±0.02	8.1±0.2	115
Pd foil	4.4±0.3	Pd-Pd	12	2.742±0.01	5.7±0.2	429
PdO	6.3±0.8	Pd-O	6	2.01±0.01	2.0±0.6	383
Catalysts						
Au	3.6±0.6	Au-Au	10.7±0.8	2.83±0.01	11.0±0.7	135
Au@Pd20% *Au-L3*	4.4±0.3	Au-Au	10.0±0.3	2.84±0.01	8.0±0.6	14
		Au-Pd	0.4±0.2	2.73±0.04		
Au@Pd20% *Pd-K*	4.4±0.3	Pd-Pd	0.8±0.3	2.75±0.03	3.5±0.1	199
		Pd-Au	4.8±0.8	2.82±0.01		
Au@Pd40% *Au-L3*	4.4±0.3	Au-Au	10.2±1.2	2.85±0.01	9.3±1.0	46
		Au-Pd	0.6±0.3	2.79±0.03		
Au@Pd40% *Pd-K*	5.1±0.2	Pd-Pd	4.6±1.0	2.75±0.01	7.7±0.9	43
		Pd-Au	2.3±0.5	2.79±0.02		
Au@Pd60% *Au-L3*	4.7±0.5	Au-Au	6.7±1.6	2.84±0.01	9.4±0.4	667
		Au-Pd	2.3±0.7	2.78±0.09		
Au@Pd60% *Pd-K*	8.3±0.7	Pd-Pd	5.3±0.9	2.73±0.01	6.7±0.7	228
		Pd-Au	1.6±0.5	2.79±0.02		
Au_2Pd_1 Au-L3	4.7±0.7	Au-Au	7.2±0.8	2.82±0.01	7.7±1.2	268
		Au-Pd	3.2±0.4	2.80±0.01		
Au_2Pd_1 Pd-K	2.4±0.4	Pd-Pd	4.5±0.4	2.78±0.01	11.0±0.3	113
		Pd-Au	6.4±0.5	2.77±0.01		

REFERENCES

(1) Fedlheim, D. L.; Foss, C. A. *Metal Nanoparticles: Synthesis, Characterization, and Applications*; Edition, 1st, Ed.; CRC Press: New York, 2001.
